# Massed treatment of posttraumatic stress disorder, traumatic brain injury, and co-occurring conditions: the Home Base intensive outpatient program for military veterans and service members

**DOI:** 10.3389/fpsyt.2024.1387186

**Published:** 2024-10-30

**Authors:** Laura K. Harward, René M. Lento, Andrew Teer, Stephanie Samph, Megan E. Parmenter, Joseph Bonvie, Charlotte Magee, Lauren Brenner, Kaitlin Picard, Wesley Sanders, William Joseph Tinney, Cyrielle Andrew, Jessica Covitz, Katrina Echevarria, Ryan Vanderweit, Nicolette S. Maggiolo, Kaloyan S. Tanev

**Affiliations:** Massachusetts General Hospital, Harvard Medical School, Boston, MA, United States

**Keywords:** military, PTSD - posttraumatic stress disorder, massed treatment, military mental health, veteran mental health, intensive outpatient program

## Abstract

The two-week Home Base Intensive Clinical Program (ICP) provides treatment to veterans and active duty service members suffering from primary diagnoses of posttraumatic stress disorder (PTSD), traumatic brain injury (TBI), anxiety, or depression. First launched in 2015, this paper provides a programmatic update, including new treatment components implemented since inception, and examines outcomes for all participants who entered the program from September 2015 to July 2024 (*n* = 2561). The Home Base ICP provides a massed care approach through daily individual Prolonged Exposure, Cognitive Processing Therapy, Unified Protocol, or cognitive rehabilitation, along with groups targeting coping skills. Participants entering the program are provided with core group programming, as well as individualized therapy sessions tailored to their unique needs and symptom presentation. Supplemental dual recovery support is also available for all participants with co-occurring substance use or behavioral addiction concerns. Participants' support people receive education, support, and case management services. Participants have a multidisciplinary team comprising therapists, psychopharmacology providers, case managers, nurses, and wellness providers. Results demonstrate that program participants exhibited statistically significant reductions in PTSD symptoms (Cohen's *d* = 0.80), depression (*d* = 0.68), post-concussion symptoms (*d* = 0.71), and increased satisfaction in social roles *(d* = -0.65). Completion rate was 94.60% (*n* = 2422), suggesting that the Home Base ICP is a well-received and effective model of care for veterans and service members.

## Introduction

Since 2001, more than 2.6 million United States service members have been deployed or served in support of conflicts in Iraq and Afghanistan. Of those, one in four experience posttraumatic stress disorder (PTSD; 1) and one in five experience traumatic brain injury (TBI; 2). Suicide rates among Army and Marine Corps personnel are double the national average (VA Suicide Prevention). Sleep disturbances, anger, hypervigilance, substance misuse, and chronic physical and mental health disorders are seen frequently in returning service members and tend to co-occur (2–4). Although evidence-based treatments exist, established outpatient treatments have not adequately met the needs of the veteran and military population due to high drop-out rates (5, 6) and difficulties accessing treatment (7, 8). One solution to these barriers has been the introduction of intensive or "massed" delivery of protocols for trauma-focused treatment (e.g., 9–11). The growing literature on massed treatments suggests reduced dropout rates (12, 13) and possibly improved treatment outcomes (13, 14).

## Context

Home Base is a Massachusetts-based nonprofit that provides free mental health services – including transportation, lodging, and meals – for military service members, veterans, and their families. The two-week Home Base Intensive Clinical Program (ICP), an intensive outpatient program, launched in 2015 to address treatment gaps for service members and veterans struggling with PTSD and TBI. Serving individuals from across the globe, the program compresses approximately seventy hours of care into two weeks (15). The program benefits from philanthropic support that enables program design that attracts participants into care and minimizes treatment barriers. Since inception, the program has served over 2,500 participants and has maintained a 94.60% completion rate. Veterans and service members served by Home Base are often affected by comorbid mental diagnoses, substance misuse, cognitive impairment, occupational difficulties, and/or marital and family stress. Accordingly, a comprehensive treatment team approach was designed to address these comorbidities in a supportive environment away from home stressors, while also providing external lodging at a hotel to offer increased autonomy. This treatment model aims to harness the camaraderie of shared experience, integrating individual and group skill-building activities to enhance resilience, self-care, and overall health and wellness.

## Program recruitment and admissions

The Home Base ICP has a multipronged recruitment strategy to reach active duty service members and veterans. These include: (1) veteran and military family outreach teams who regularly connect with regional military organizations and participate in community events (16); (2) engagement with partner sites and other benevolence organizations; (3) networking with local and national mental health organizations and clinics who serve military communities, as well as participation in relevant conferences; (4) advertisement through social media and the Home Base website; and (5) word-of-mouth referrals from program alumni. Over the years, Home Base staff have fostered partnerships with community organizations that represent underserved populations such as Native American, Hispanic, and women veterans, working closely with these collaborators to advise on enhancements to typical program structure that further reduce barriers to engagement (e.g., translation of materials into Spanish, offering all-female cohorts).

The admissions process begins with the prospective participant submitting a self-referral form and a reason for seeking services. Any service member or veteran seeking treatment for symptoms of PTSD, TBI, anxiety, depression, or prolonged grief is considered, regardless of service era, branch of service, discharge status, or deployment history. Relative psychosocial stability, including stable housing, is required to set participants up for continued success following program completion.

The prospective participant is contacted to schedule a screening that includes demographic information, military history, medical history, and clinical history to include current symptoms, risk concerns, substance use, a TBI screen, and current treatment. Pre-treatment measures, including the PTSD Checklist for DSM-5 (PCL-5; 17) and the Patient Health Questionnaire (PHQ-9; 18), as well as releases of information for past treatment or current providers, are then sent to the prospective participant.

Upon receipt, records are reviewed by both medical and psychological team members to (a) identify potential concerns that may impact care or require accommodation during the Home Base ICP; and (b) confirm assignment to a Mental Health, TBI, or Hybrid treatment track. If no concerns are identified during this process, the participant is contacted by a social worker to discuss scheduling. If concerns do arise, they are discussed within a multidisciplinary admissions team and the prospective participant is provided with treatment recommendations. Examples of possible concerns include serious medical impairment, cognitive impairment, and/or other psychiatric conditions requiring higher level of care. Individuals are not eligible for the program if they present with active symptoms of psychosis or mania, acute behavioral concerns that require resolution (*e.g.* recent arrests) and/or would disrupt the program milieu beyond what is expected for this population (e.g., recent instance of violence toward other patients or providers), suicide or homicide attempt within the last 90 days, psychiatric hospitalization within the last 30 days, or substance withdrawal risk requiring detoxification. Potential participants must also be willing to abstain from illicit substances and alcohol during the program. If a veteran or service member previously attended the program, repeating the program requires a review of adherence to previous discharge recommendations and rationale for the likelihood of a better outcome following a second course of treatment.

## Assignment to treatment track

During the admissions process, the prospective participant is assigned to the Mental Health track, TBI track, or a Hybrid track based upon presenting symptoms, history of TBI, and treatment resource availability in the patient's community. For individuals who present with both mental health and TBI concerns, the following criteria are used to determine track assignment: (a) whether mental health or TBI predominantly impacts the patient's current functioning; (b) the individual's preference; (c) the individual's history of evaluation and care pertaining to each condition; and (d) the availability of local care resources in the patient's community. For example, if an individual has limited access to both mental health and TBI-related services, Hybrid track is often recommended to maximize mental health and cognitive health gains from our program and to inform treatment planning after the completion of our program. In summary, if an individual has primary needs, they are assigned to the appropriate track; if an individual has both mental health and TBI needs, they are assigned to the Hybrid track; if access to a specific mode of treatment (*e.g.* cognitive rehabilitation services) is limited, they may be assigned to the track that provides the mode of treatment lacking in the patient's community. And, if further assessment is needed to clarify track assignment, the prospective patient is offered a pre-program multiday evaluation by a Home Base team, which is described in further detail below.

## Key programmatic elements and treatment interventions

The Home Base ICP model previously discussed by Harvey and colleagues (2019), has undergone rapid expansion since 2018, both in terms of number of participants served and in treatment options provided. See **Table 1** for a summary of the key programmatic elements that have been added or modified since the original publication. The program uses a whole-health-based approach, staffed by a multidisciplinary team comprising psychiatrists, nurse practitioners, clinical psychologists, neuropsychologists, clinical social workers, speech language pathologists, nurses, physical medicine and rehabilitation (PMR) specialists, registered dieticians, integrative health providers, and peer-to-peer veteran and family support specialists. Participants are assigned to a primary Mental Health track (primarily PTSD, anxiety, and/or depression), TBI, or Hybrid MH-TBI track during the admissions process after identification of their current symptomatology, history of TBI, and primary presenting concerns. Upon arrival, they receive daily evidence-based individual therapy and skills-focused group therapies grounded in CBT approaches. Additionally, all participants are provided psychopharmacology and case management, and engage in health and wellness programming described herein. Additional consultation sessions are also available based on the participant's needs, and support person programming is offered for participants and their loved ones. Further, all programming is supported and informed by veteran peers on staff who serve as liaisons between participants and providers. See **Table 2** for services provided by treatment track.

**Table 1:** Summary of Home Base Intensive Clinical Program enhancements to key programmatic elements since 2018.

	15	Present
**General Enhancements**	Served post 9/11 veterans and active duty service members	Serves all era veterans and service members
	Single cohort: 8-10 participants	Double cohorts: 10-12 participants per cohort
	Cohorts run approximately once per month	Cohorts run approximately twice per month
	PTSD or TBI tracks	PTSD, TBI, or Hybrid tracks
	No Dual Recovery Supplement	Optional Dual Recovery Supplement
	No direct line of contact to Veterans Affairs (VA)	In-house VA liaison available to meet with all patients one-on-one
**Individual Therapy**	PE or CPT	PE, CPT, or UP
**Group Therapy**	4 SUD Education sessions for select participants	1 SUD Education session for all participants
	No process groups	No process groups
**Adjunctive Care Elements**	No formal consult system	*Ad-hoc* consults for substance use, behavioral addiction, sleep, parenting, cognitive rehabilitation
	In-person participation from support person	Virtual participation from support person(s)

PTSD, posttraumatic stress disorder; TBI, traumatic brain injury. PE, Prolonged Exposure; CPT, Cognitive Processing Therapy; UP, Unified Protocol. SUD, substance use disorder.

**Table 2:** Current services provided by treatment track.

Service	Mental Health Track	TBI Track	Hybrid Track
Skills Groups			
Resilient Warrior, DBT Skills, Warrior Cognitive Health, *In Vivo*, Processing Group, SUD Education	✓	✓	✓
Wellness Programming			
Mindful Movement, Foundations of Fitness, Expressive Art Therapies, Nutrition	✓	✓	✓
Individual Therapy			
Prolonged Exposure Therapy	✓		✓
Cognitive Processing Therapy	✓		✓
Unified Protocol	✓		✓
Cognitive Rehabilitation		✓	✓
Individual Consultive Services			
SUD, Parenting, Sleep, Cognition	✓	✓	✓
**Case Management**	✓	✓	✓
**Psychopharmacology**	✓	✓	✓
**Physical Medicine & Rehabilitation**		✓	✓
**Physical Therapy**		✓	✓
**Support Person Programming**	✓	✓	✓
**Peer Support**	✓	✓	✓
**Recreational Activities**	✓	✓	✓

## Mental health track

As described above, participants are assigned to the Mental Health track prior to arrival and complete a clinical interview and eight 60-minute sessions of daily individual therapy provided by a psychologist or clinical social worker. Given the growing evidence for the efficacy (10, 15, 19–21) and tolerability (11, 22, 23) of massed trauma-focused treatments, treatment primarily consists of Prolonged Exposure (PE) or Cognitive Processing Therapy (CPT), two of the gold-standard treatments for PTSD. In line with best practices, shared decision making helps to determine treatment intervention after completion of the clinical interview. Participants who present with psychiatric diagnoses other than PTSD (e.g., persistent complex bereavement disorder, major depressive disorder) often engage in other evidence-based treatment modalities, including Prolonged Grief Treatment (24), cognitive behavioral therapy, or an adaptation of the Unified Protocol (UP; 25) designed for the Home Base ICP in collaboration with the UP Institute.

## TBI track

Participants may be assigned to the TBI track at any time during the admissions process and, if indicated, undergo a pre-program multiday evaluation by a Home Base team including PMR, physical therapy, neuropsychology, psychiatry, and psychology. These evaluations provide treatment recommendations for the Home Base ICP, and treatment determinations are made through collaborative decision making with the participant. Potential outcomes include referral to one of the program tracks or to local providers.

Participants in the TBI track receive daily, 60-minute individual cognitive rehabilitation sessions administered by a licensed speech-language pathologist or neuropsychologist. Our cognitive rehabilitation model is based on cognitive compensation, which focuses on the use of internal and external strategies to support cognition (26). Individual session treatment goals are determined collaboratively, and participants practice compensatory strategies aimed at improving daily cognitive functioning. Additionally, participants are offered three to four vestibular physical therapy sessions, and consultation sessions with a PMR physician for care of medical symptoms such as headaches. Based on individual needs, participants may be referred to specialized evaluations (*e.g.* audiology, neuroendocrinology, sleep medicine, orthopedics).

## Hybrid track

Given the relationship between mental health conditions and TBI in military personnel (27), the Hybrid track was developed for participants who would benefit from engaging in services offered within both the TBI and mental health tracks. A meta-analysis has shown that cognitive rehabilitation is effective for improving memory in participants with PTSD (28); conversely, other studies have shown that PTSD treatments are effective regardless of the presence of TBI or its severity (29). Accordingly, a treatment plan may consist of a combination of vestibular therapy, individual psychological therapy, and PMR consultation to help address each patient's specific sequela.

## Group therapy interventions

**Figure 1:**
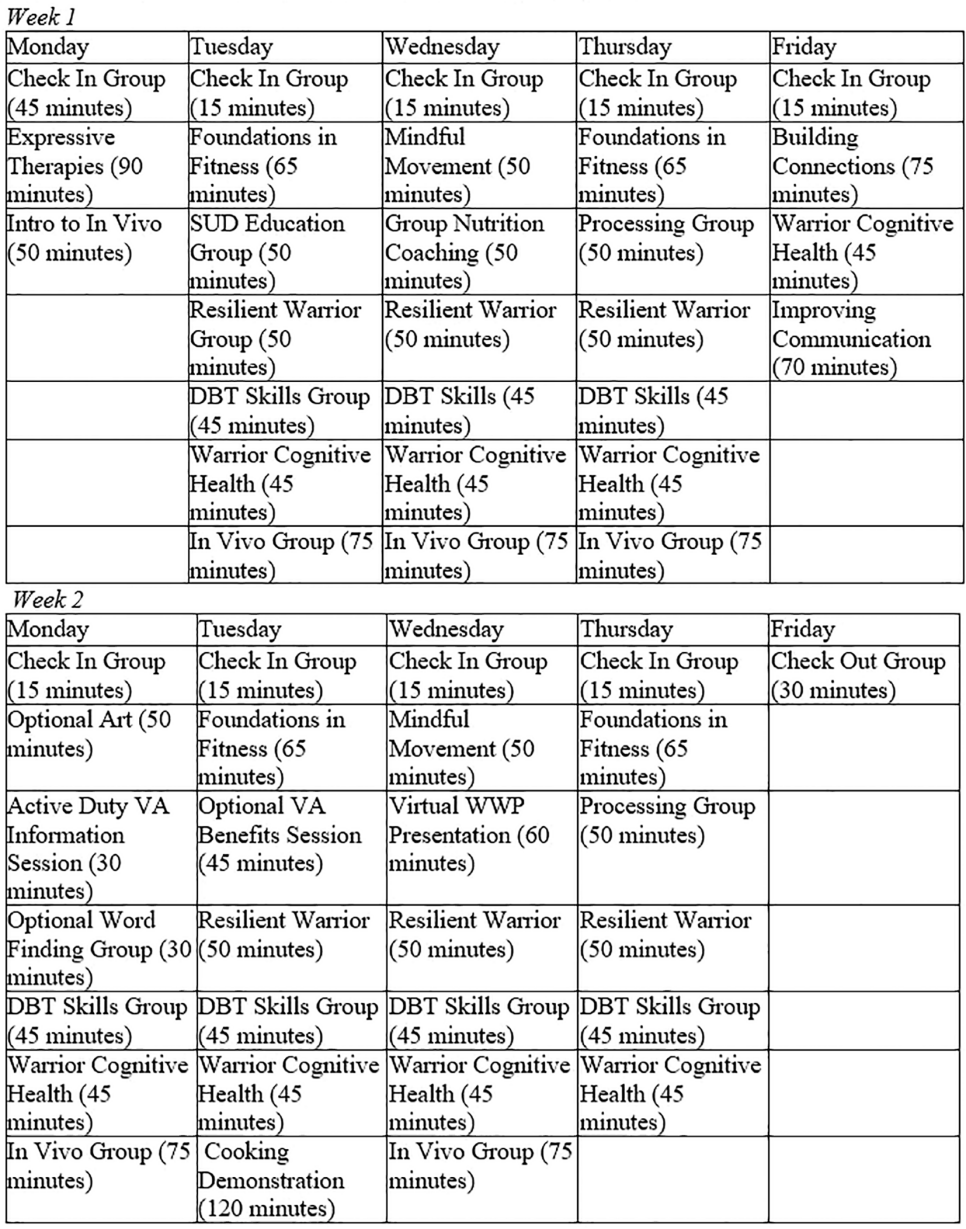
Sample group schedule for participants on the mental health, TBI, or hybrid tracks.

All participants receive approximately 45 hours of group therapy. Groups include dialectical behavioral therapy (DBT; 30) skills, *in vivo* exposure adapted from PE (9), cognitive health, Resilient Warrior adapted from the Relaxation Response Resiliency Program (31, 32), substance use education, and process groups. This model was selected to facilitate group cohesion and to increase participants' healthy coping while participating in daily individual therapy (33). A sample group schedule is provided in **Figure 1**.

DBT skills group target deficits in mindfulness, emotion regulation, distress tolerance, and interpersonal functioning commonly seen in PTSD and other emotional disorders (34, 35); *in vivo* exposures target avoidance that interferes with extinction learning and self-efficacy (36); and Resilient Warrior sessions target the development of coping skills for distress through mind-body techniques (37). Cognitive health and substance use education groups provide education and coping skills to address the cognitive impairments and substance use concerns commonly reported in military and veteran populations (38, 39).

## Psychopharmacology

Every participant is assigned a psychopharmacology provider (psychiatrist or psychiatric nurse practitioner). This plan may include psychoeducation, adjustments to existing medications, or non-pharmacologic interventions. Psychopharmacology providers adhere to treatment guidelines for PTSD, depression, and anxiety (*e.g.* 40), which do not support prescribing benzodiazepines. Participant preferences regarding medications are respected, with some willing to initiate pharmacotherapy, and others hesitant or opposed to due to stigma, side effects, potential negative implications to their military career, or prior medication trials. Psychopharmacology providers meet with participants as frequently as clinically indicated to address these concerns, make recommendations for ongoing care at home, answer questions, and collaborate with the participant's local treatment team.

## Case management

Case management is provided to all participants, many of whom live out of state or far from large Veterans Affairs (VA) medical centers. A social work or nurse case manager works closely with each participant and their support person to ensure a smooth transition both into and out of the program. The case manager meets with the participant to assess and address psychosocial concerns (e.g., housing, financial stress) or treatment needs, and collaborates with support people and other providers to determine an individualized discharge plan. One month after completion, a resource specialist calls graduates of the program to assess needs and inquire about additional resource requests. Any responses that indicate that the individual is in need of resources are directed back to their Home Base ICP case manager for follow-up.

Through a Memorandum of Understanding (MOU) between Home Base and the VA, a VA social worker is embedded in Home Base to assist with records transfer, benefits support, and connection back to the VA upon program completion. This VA liaison meets with participants during the program to discuss any referrals made by Home Base to the participant's community VA Medical Center or Community Based Outpatient Clinic.

## Wellness programming

### Art therapy

The expressive art therapy groups use art media and the creative process to explore feelings, reduce stress and anxiety, foster self-awareness, improve communication skills, and promote a sense of community (41). Participants engage in two group sessions and have access to optional individual consultations and drop-in time.

### Fitness

**Figure 2:**
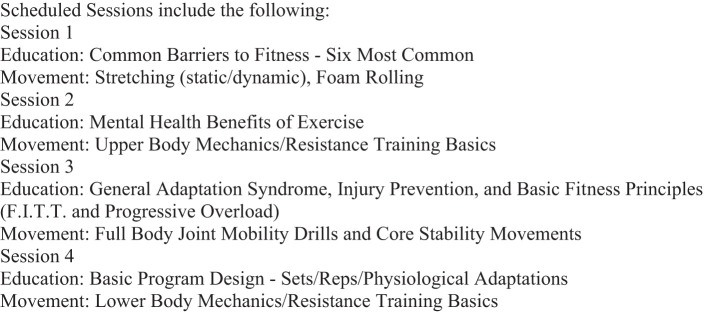
Sample schedule fitness.

Fitness sessions were designed to improve participants' experiential understanding of the benefits of physical health on mental health and overall well-being. They comprise four, 60-minute sessions with a certified strength and conditioning specialist and focus on foundational movements and education (see **Figure 2** for sample schedule). Additionally, individual consultations are available upon request, as are open gym times in the clinic's fitness center.

### Nutrition

Registered dietitians provide evidence-based guidance on food and nutrition through individual counseling, group classes, and hands-on cooking. Topics discussed include tactical athlete performance optimization, chronic disease prevention and management, weight management, mental health, and mindful eating. Group classes explore the intersection between nutrition and mental health, often a new experience for participants. Educational content includes strategies for fostering ideal gut health, improving mealtime balance, maintaining greater consistency, and supporting healthy relationships with food. Participants prepare a meal together during the program, which encourages participants to learn new skills, discuss the nutritional benefits of food, and promotes social connection.

## Dual recovery supplement

Substance use disorders (SUDs) are commonly comorbid with PTSD and TBI (42, 43), with some studies evincing a rate of around 50% (e.g. 44). Participants frequently request assistance with reducing or eliminating use; thus, the voluntary Dual Recovery (DR) supplement was launched in 2021 to increase access to, and successful completion of, the program for veterans and service members who are in early recovery or managing substance misuse. In addition to the standard program components, individuals experiencing substance misuse or behavioral addiction meet with a SUD or behavioral addiction clinician for an educational meeting the week prior to arrival, and for up to six additional individual therapy sessions. These sessions incorporate principles from Motivational Interviewing (45) and cognitive-behavioral strategies from the Concurrent Treatment of PTSD and Substance Use Disorders Using Prolonged Exposure (COPE; 46) protocol. A Recovery Coach is available to all participants during the program and provides caring contact emails to DR participants for one year following program completion. Further, DR participants are matched with medication providers trained in addiction medicine, who incorporate medications for substance use as clinically indicated. Commonly prescribed medications include FDA-approved medications for alcohol use disorder and nicotine use disorder. Gabapentin is also used to manage cannabis withdrawal symptoms. Although participants are not started on buprenorphine or methadone during the program, providers can support participants in continuing established treatment.

## Consultation services

### SUD and compulsive behaviors

Alongside implementation of the DR supplement, the need exists for addiction-related support for participants with milder presentations. A consult service for substance misuse and/or behavioral concerns (e.g., excessive shopping, videogaming, pornography, or gambling) entails one to three therapy sessions tailored to assess and triage the unique needs of the participant. Consults are available for (a) participants identified during the admissions process who could benefit from support but do not want and/or need the full DR supplement; (b) participants identified during the admissions process who are lower risk or cannot be accommodated into the full supplement due to logistical limitations; or (c) participants for whom addiction issues become apparent after program start.

### Sleep

As many as 80-90% of individuals with PTSD report symptoms of insomnia (47). Participants who identify improving sleep as a treatment goal can schedule sleep consults with providers trained in cognitive behavioral therapy for insomnia (CBT-I). These consults are tailored to participants' individual needs and consist of an assessment of current sleep difficulties, psychoeducation about sleep processes and factors that perpetuate insomnia, and review of sleep hygiene tools. Providers often incorporate additional information from CBT-I, including information on sleep diaries and sleep restriction.

### Parenting

Parenting consultative services are available based on need, presentation, and interest. Tailored to the presenting concern, psychoeducation is provided in three domains: normative developmental expectations; resources or skills that directly address parenting behavior (e.g., setting consistent expectations in the home); and considerations regarding the impact of PTSD on caregiving. Parenting support incorporates a cultural humility framework, recognizing and discussing the impact of military culture and belief systems on the participant's perceptions and expectations about parenting. Additional time in session is devoted to referrals including family therapy, parenting support, and couples therapy in the home.

### Cognition

Consistent with studies that suggest cognitive symptoms are pervasive in veterans with PTSD and depression (48), participants on the mental health track frequently report cognitive concerns. These participants may be referred for ancillary cognitive consultations to receive individualized practice of strategies reviewed in the Cognitive Health group.

## Support person programming

Individuals close to participants are often impacted by issues arising from their military service, reintegration, mental health concerns, and TBI symptoms. Limited resources are available for support people and there is limited expertise among community providers providing such services. Research has demonstrated that support persons can increase motivation for treatment (49) and reinforce treatment outcomes and adherence (50). Participants can invite a support person to participate in two days of educational programming. The program provides a supportive and validating environment for support people to share their own experiences and struggles while also indirectly supporting the participant's recovery.

Support people receive 11 hours of group-based programming, including psychoeducation covering PTSD, readjustment issues, cognitive health, substance use, and the impact of trauma on relationships, as well as overviews of programming (e.g., integrative wellness, DBT skills). Two conjoined participant-support virtual groups also offer opportunities to practice effective communication skills. Support people connect with social workers who offer case management resources for care in their community as needed.

## Peer support

Veteran Outreach Coordinators (VOCs) are veterans and service members who provide non-clinical peer support services to participants. They work closely with clinical leads to inform programmatic planning, provide insight into military culture, and ensure appropriate tailoring of the services to the participant population. VOCs actively listen to and advocate for participants throughout their treatment, aiming to promote a treatment milieu that offers psychological safety and mutual respect.

Consistent with established literature (51, 52), participants are more willing to speak to fellow veterans about their service and disclose uncomfortable matters to those with similar lived experience. This information allows for rapid response within the clinical team to better triage risk and treatment-related concerns, and to inform culturally sensitive treatment plans. VOCs aim to redefine veteran peer relationships for participants who may have had a difficult experience during their time in service. This connection aims to help veterans build long-lasting relationships within their community.

The VOC team oversees logistics for program arrivals, daily check-ins, and non-clinical evening and weekend programming. Non-clinical activities provide opportunities for participant cohesion, expansion of support networks, and practicing skills taught during clinical programming outside the clinical setting. Evening programming typically involves military history activities; weekend programming focuses on activities that foster communication skill development and time for decompression.

## Methods and analyses

### Participant sample

Participants were 2561 veterans and active duty service members who participated in the Home Base ICP between September 2015 to July 2024. This program period included 261 cohorts of 6-14 participants referred from 50 states and 13 jurisdictions.

### Measures

Participants complete a set of clinical measures at pre-treatment and post-treatment. Measures assess key treatment targets of PTSD, depression, neurobehavioral symptoms, and satisfaction with their ability to participate in expected social roles. Primary measures of symptom improvement include the PTSD Checklist for DSM-5 (PCL-5; 17), the Patient Health Questionnaire (PHQ-9; 18), and the Neurobehavioral Symptom Inventory (53). A Patient-Reported Outcomes Measurement Information System (PROMIS) measure was used to assess satisfaction with social roles, ability to participate in social roles (54, 55), and self-efficacy with cognitive symptom management (56).

### PTSD symptoms

The PCL-5 is a 20-item, self-report measure that assesses the 20 *DSM-5* symptoms of PTSD. It has been shown to be a psychometrically sound measure for identifying a provisional PTSD diagnostic status, quantifying PTSD symptom severity, and detecting clinical change among treatment-seeking military service members (57). For each item, respondents report the degree to which they have been bothered by a symptom on a 5-point scale ranging from 0 = *not at all* to 4 = *extremely*. Scores range from 0 to 80 with higher scores indicating greater severity of PTSD. The "past month" version was used at pre-treatment and the "past week" version was used at post-treatment. Research suggests that a PCL-5 cutoff score between 31-33 is optimally efficient for diagnosing PTSD (58). According to the National Center for PTSD (59), PCL-5 change scores of 10-20 likely represent clinically significant change. Cronbach's alpha in our sample was 0.94 pre-treatment and 0.96 post-treatment.

### Depressive symptoms

The PHQ-9 is a 9-item, self-report measure of depression based on the *DSM-IV-TR* symptoms of depression. The PHQ-9 is widely used in primary care and psychiatric settings and shown to be reliable and valid when screening depression in those with TBI (60). For each item, respondents report the degree to which they have been bothered by a symptom over the past two weeks on a 4-point scale ranging from 0 = *not at all* to 3 = *nearly every day.* Total scores range from 0 to 27, with increased scores reflecting greater symptom severity. The alpha in our sample was 0.85 before treatment and 0.89 after treatment.

### Neurobehavioral symptoms

The Neurobehavioral Symptom Inventory (NSI) is a 22-item symptom rating scale used in the U.S. Department of Defense and Veterans Administration to measures post-concussion symptoms following TBI. The scale includes a broad range of symptoms such as headaches, dizziness, fatigue, sleep difficulties, anxiety, irritability, and cognitive deficits. Structural equation modeling has repeatedly demonstrated that the NSI is multidimensional, measuring a range of somatic/sensory, affective, and cognitive symptoms in those with TBI or due to other causes, including PTSD (61–63). For each item, respondents report the degree to which they have been disturbed in the past two weeks on a 5-point scale ranging from 0 = *none* to 4 = *very severe*. Total scores range from 0 to 80. Cognitive subscale severity scores are calculated by summing items 13-16. The alpha in our sample was 0.92 before treatment and 0.94 after treatment on the NSI total score and 0.89 before treatment and 0.91 after treatment for the NSI cognitive subscale.

### Patient-reported outcomes

The Satisfaction with Participation in Social Roles–Short Form 8a (PROMIS Satisfaction) is an 8-item measure used to assess satisfaction with participation in different social settings such as work, family, leisure activities, and relationships with friends. For each item, respondents report the degree of satisfaction over the past 7 days on a 5-point scale ranging from 0 = *not at all* to 4 = *very much*. The pre- and post-treatment alphas for this measure were 0.94 and 0.96, respectively.

The PROMIS Ability to Participate in Social Roles and Activities– Short Form 4a (PROMIS Ability) is a 4-item measure consisting of a list of statements about participating in activities with family and friends. Each item is ranked on a 5-point scale ranging from 0 = *never* to 5 = *always*. Cronbach's alpha was 0.89 before treatment and 0.92 after treatment.

The PROMIS Self-Efficacy for Management of Chronic Conditions– Short Form 4a (PROMIS Self-Efficacy) is a 4-item measure consisting of a list of statements asking for participants' confidence in doing certain activities. Each item is ranked on a 10-point scale ranging from 1 = *not at all* to 10 = *totally confident*. The alpha in our sample was 0.94 pre-treatment and 0.95 post-treatment.

### COVID-19 treatment adjustments

Following a four-month hiatus during COVID-19, the program reopened in July 2020. All individual-based interventions remained virtual via telemedicine, along with art therapy, fitness, and yoga. Groups therapies were offered in person with the intent to build comradery and cohesiveness. In August 2022, the remaining virtual interventions all shifted to in person.

### Statistical approach

This report examined outcomes for all participants who entered the program from September 2015 to July 2024 *(N* = 2561). Data from participants who did not complete the program due to early departure or discharge (*n* = 139) were not included in statistical analysis. For each measure, if data were missing at either baseline or endpoint, the participant was excluded from that analysis. Missing data is attributed to a participant not completing the program or opting out of completing their self-report measures. A mixed repeated measures ANOVA was conducted to analyze pre- and post-treatment scores for all measures and to compare measure score changes across all three treatment tracks (mental health, TBI, hybrid). Descriptive statistics were generated for PCL-5 and PHQ-9 scores at all timepoints, for which all available data from participants who completed the program were considered. Additionally, one-way ANOVA tests were conducted to determine if gender, race, or primary treatment type influenced pre- to post-program measure score changes for PCL-5 and PHQ-9. Games-Howell *post-hoc* tests were later conducted to analyze significant results. One-way ANOVA tests were also conducted to compare pre-treatment to post-treatment measure score changes across pre-, during-, and post-COVID (September 2015-February 2020, March 2020-August 2022, September 2022-July 2024, respectively) to determine if COVID-era treatment impacted treatment measure outcomes Pre- and post-program measure score changes for PCL-5 and PHQ-9 were additionally analyzed by independent samples *t*-tests, for which pre-post change effect sizes were also calculated. A linear regression was performed to determine if the age of the patient predicted measure score changes for PCL-5 and PHQ-9. The level of statistical significance was set to *p* = 0.05 (two-tailed). All statistical analyses were conducted using SPSS version 28.

### Results sample description

Participants had a mean age of 46.5 years (*SD* = 9.3). One fifth (19.9%) were active duty service members while the majority (75.8%) were prior service military personnel who had separated from service. The Army was the most represented among the military branches (61.2%). Additional demographic data are presented in **Table 3**. Of all participants, 88% sought care or evaluation for PTSD, 9.5% sought care or evaluation for TBI, and 2.5% sought care or evaluation for both PTSD and TBI through the Hybrid track. A subset of 9.5% participated in supplemental Dual Recovery sessions. Of the participants who reported a past diagnosis (*n* = 1231), 83.3% (*n* = 1025) reported more than one diagnosis, with PTSD (81.9%, *n* = 1008) being the most reported previous diagnosis.

**Table 3:** Demographic characteristics of N=2560 Intensive Clinical Program Participants.

Characteristic	Mental Health *N* (%)	TBI *N (%)*	Hybrid *N (%)*
Gender (*n* = 2560):			
** Male**	1814 (80.51)	215 (88.8)	58 (89.2)
** Female**	434 (19.26)	26 (10.7)	7 (10.8)
** Other**	5 (0.22)	1 (0.4)	–
**Heterosexual (*n* = 2050)**	1608 (92.52)	233 (93.95)	–
Race (*n* = 2531)			
** American Indian/Alaska Native**	14 (0.6)	1 (0.4)	1 (1.6)
** Asian**	39 (1.8)	4 (1.7)	2 (3.1)
** Black/African American**	251 (11.3)	19 (7.9)	1 (7.7)
** Native Hawaiian/ Pacific Islander**	22 (1.0)	4 (1.7)	–
** More than One Race**	59 (2.6)	4 (1.7)	2 (3.1)
** White**	1678 (74.4)	198 (81.8)	50 (78.1)
** Other**	140 (6.3)	12 (5.0)	3 (4.7)
**Hispanic/Latino (*n* = 2525)**	303 (13.7)	24 (9.9)	9 (14.1)
Relationship Status (*n* = 2511)			
** Divorced**	306 (13.9)	25 (10.3)	10 (15.9)
** Engaged**	26 (1.2)	4 (1.7)	2 (3.2)
** Married/Domestic Partnership**	1210 (59.4)	166 (68.6)	39 (61.9)
** Single**	386 (17.5)	30 (12.4)	9 (14.3)
** Separated**	133 (6.0)	12 (5.0)	3 (4.8)
** Other**	44 (2.0)	5 (2.0)	–
Military Branch (*n* = 2529)			
** Army (including Reserves and National Guard)**	1196 (53.7)	117 (48.3)	32 (52.5)
** Air Force (including Reserves and National Guard)**	276 (12.4)	24 (9.9)	3 (4.9)
** Coast Guard (including Reserves)**	35 (1.6)	1 (0.4)	2 (3.3)
** Marine Corps (including Reserves)**	264 (11.9)	31 (12.8)	6 (9.8)
** Navy (including Reserves)**	455 (20.4)	69 (28.5)	18 (29.5)
Military Status (*n* = 2516)			
** Active Duty**	393 (17.8)	83 (34.6)	24 (37.5)
** Discharged**	1010 (45.7)	72 (30.0)	23 (35.9)
** Inactive**	3 (0.1)	1 (0.4)	1 (1.6)
** Medically Retired**	345 (15.6)	42 (17.5)	9 (14.1)
** National Guard**	55 (2.5)	2 (0.8)	–
** Reserves**	26 (1.2)	4 (1.7)	–
** Retired**	363 (16.4)	35 (14.6)	6 (9.4)
** Not Applicable**	17 (0.8)	1 (0.4)	1 (1.6)
Previous Diagnoses (*n* = 1231)			
** PTSD**	910 (40.4)	89 (36.8)	9 (13.8)
** TBI**	392 (17.4)	92 (38.0)	9 (13.8)
** Anxiety**	795 (35.3)	94 (38.8)	12 (18.5)
** Depression**	788 (35.0)	85 (35.1)	13 (20.0)
** Substance Use Disorder (Alcohol)**	180 (8.0)	10 (4.1)	–

### Program completion

A total of 2561participants attended the treatment program with a 94.6% completion rate (*n* = 2422). Within the individual treatment tracks, 94.06% (*n* = 1822) completed the mental health track and 95.83% (*n* = 253) completed the TBI or Hybrid tracks. 93.3% (*n* = 181) of participants who sought additional treatment for substance use completed the Dual Recovery track (of which 170 participants were in the mental health track and 11 were in the TBI or Hybrid track).

### Treatment outcomes

**Figure 3:**
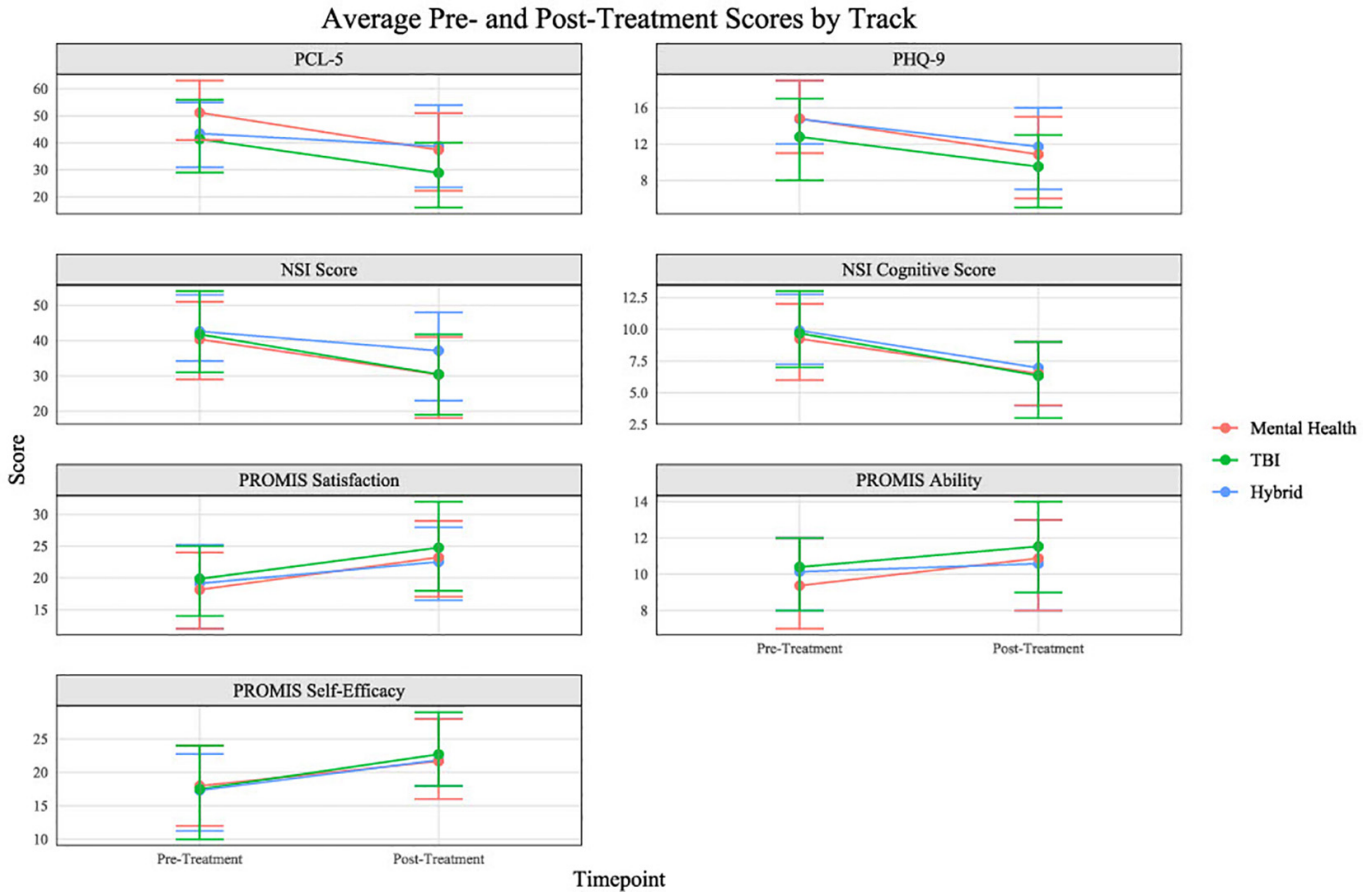
Average pre- to post-treatment measure score changes by track.

A mixed effects repeated measures ANOVA test conducted to analyze pre- to post-treatment measure scores among the three treatment tracks (MH, TBI, Hybrid) revealed significant pre- to post-treatment changes. The analysis revealed statistically significant decreases in pre- to post-treatment PCL-5, PHQ-9, NSI total, and NSI cognitive scores, and statistically significant increases in PROMIS Ability, PROMIS Self-Efficacy, and PROMIS Satisfaction scores (see **Table 4**; **Figure 3**). The analysis additionally revealed a main effect of treatment track on measure scores for the PCL-5 (*F*[2, 2069] = 7.05, *p* < 0.001), the PROMIS Ability (*F*[2, 2139] = 4.17, *p* = 0.016), the PROMIS Satisfaction *(F*[2, 2126] = 2.663, *p* = 0.07), and the PHQ-9 *(F*[2, 8.68] = *p* < 0.001). A Games-Howell *post-hoc* test indicated that patients in the MH track experienced significantly higher average PCL-5 scores (M = 8.48, *p* < 0.001) than those in the TBI track, and that those in the MH track experienced a significantly greater decrease between pre- and post-treatment PCL-5 scores (*F*[2, 2069] = 7.05, *p* < 0.001) than those in the hybrid track (MH: Mpre = 50.89, Mpost = 37.29, Mchange = -13.6; TBI: Mpre = 42.11, Mpost = 29.12, Mchange = -12.99; Hybrid: Mpre = 42.10, Mpost = 37.29, Mchange = -4.81). Tukey's *post-hoc* tests were conducted to further analyze the effect of treatment track on measure scores for the PROMIS Ability, PROMIS Satisfaction, and PHQ-9. Patients in the MH track reported significantly lower PROMIS Ability scores (M = -0.79, *p* < 0.001) compared to those in the TBI track but a significantly greater increase from pre- to post-treatment than those in the hybrid track (MH: Mpre = 9.41, Mpost = 10.87, Mchange = 1.46; TBI: Mpre = 10.34, Mpost = 11.51, Mchange = 1.17; Hybrid: Mpre = 10.32, Mpost = 10.58, Mchange = 0.26). Additionally, patients in the TBI track reported higher average PROMIS Satisfaction scores (M = 1.5, *p* = 0.4) than those in the MH track, and both MH (M = 2.55, *p* = 0.011) and TBI (M = 2.52, *p* = 0.033) patients experienced greater score improvements than those in the hybrid track (MH: Mpre = 18.15, Mpost = 23.29, Mchange = 5.14; TBI: Mpre = 19.67, Mpost = 24.77, Mchange = 5.1; Hybrid: Mpre = 19.94, Mpost = 22.53, Mchange = 2.59). Tukey's *post hoc* test also revealed that patients in the MH track had significantly higher average PHQ-9 scores (M = 1.56, *p <* 0.001) than those in the TBI track, but no significant difference in pre- to post-treatment score changes across tracks (MH: Mpre = 14.76, Mpost = 10.83, Mchange = -3.93; TBI: Mpre = 13.00, Mpost = 9.47, Mchange = -3.53; Hybrid: Mpre = 14.33, Mpost = 11.73, Mchange = -2.6). The ANOVA test additionally found a significant effect of treatment track on NSI total score change (*F*[2, 2007] = 4.49, *p* = 0.011), with Tukey's *post hoc* test revealing a significantly greater improvement in NSI scores in the TBI track (M = 6.05, *p =* 0.011) than in the hybrid track (MH: Mpre = 39.98, Mpost = 30.28, Mchange = -9.7; TBI: Mpre = 42.27, Mpost = 30.64, Mchange = -11.85; Hybrid: Mpre = 42.27, Mpost = 36.46, Mchange = -5.81).

**Table 4:** Mixed-effects repeated-measures ANOVA results for pre- to post-treatment measure outcomes and treatment track.

Measure	Effects	ANOVA			Pre-Treatment	Post-Treatment
		*F (df1, df2)*	*p*	η_p_^2^	*M (SD)*	*M (SD)*
PCL-5	Time	143.53 (1, 2069)	< 0.001	0.065	50.06 (16.02)	36.56 (18.49)
	Track	31.53 (2, 2069)	< 0.001^a^	0.03		
	Time x Track	7.05 (2, 2069)	< 0.001^b^	0.007		
PHQ-9	Time	124.5 (1, 2120)	< 0.001	0.055	14.63 (5.77)	10.75 (6.07)
	Track	8.687 (2, 2120)	< 0.001^a^	0.008		
	Time x Track	1.71 (2, 2120)	0.181	0.002		
NSI Score	Time	159.98 (1, 2007)	< 0.001	0.074	40.53 (15.35)	30.53 (16.73)
	Track	2.93 (2, 2007)	0.054	0.003		
	Time x Track	4.49 (2, 2007)	0.011	0.004		
NSI Cognitive Score	Time	74.17 (1, 754)	< 0.001	0.09	9.31 (4.15)	6.5 (4.12)
	Track	0.35 (2, 754)	0.71	0.001		
	Time x Track	0.74 (2, 754)	0.48	0.002		
PROMIS Satisfaction	Time	109.48 (1, 2126)	< 0.001	0.049	18.33 (7.31)	23.38 (7.91)
	Track	5.17 (2, 2126)	0.006^a^	0.005		
	Time x Track	2.66 (2, 2126)	0.07^a,c^	0.002		
PROMIS Ability	Time	34.02 (1, 2139)	< 0.001	0.016	9.49 (3.33)	10.94 (3.55)
	Track	6.59 (2, 2139)	0.001^a^	0.006		
	Time x Track	4.17 (2, 2139)	0.016^b^	0.004		
PROMIS Self-Efficacy	Time	69.74 (1, 2087)	< 0.001	0.032	17.92 (8.54)	21.78 (8.36)
	Track	0.191 (2, 2087)	0.826	0.000		
	Time x Track	2.45 (2, 2087)	0.086	0.002		

a = significant difference at 0.05 level between MH and TBI tracks; b = significant difference at 0.05 level between MH and hybrid tracks; c = significant difference at 0.05 level between hybrid and TBI tracks.

**Figure 4:**
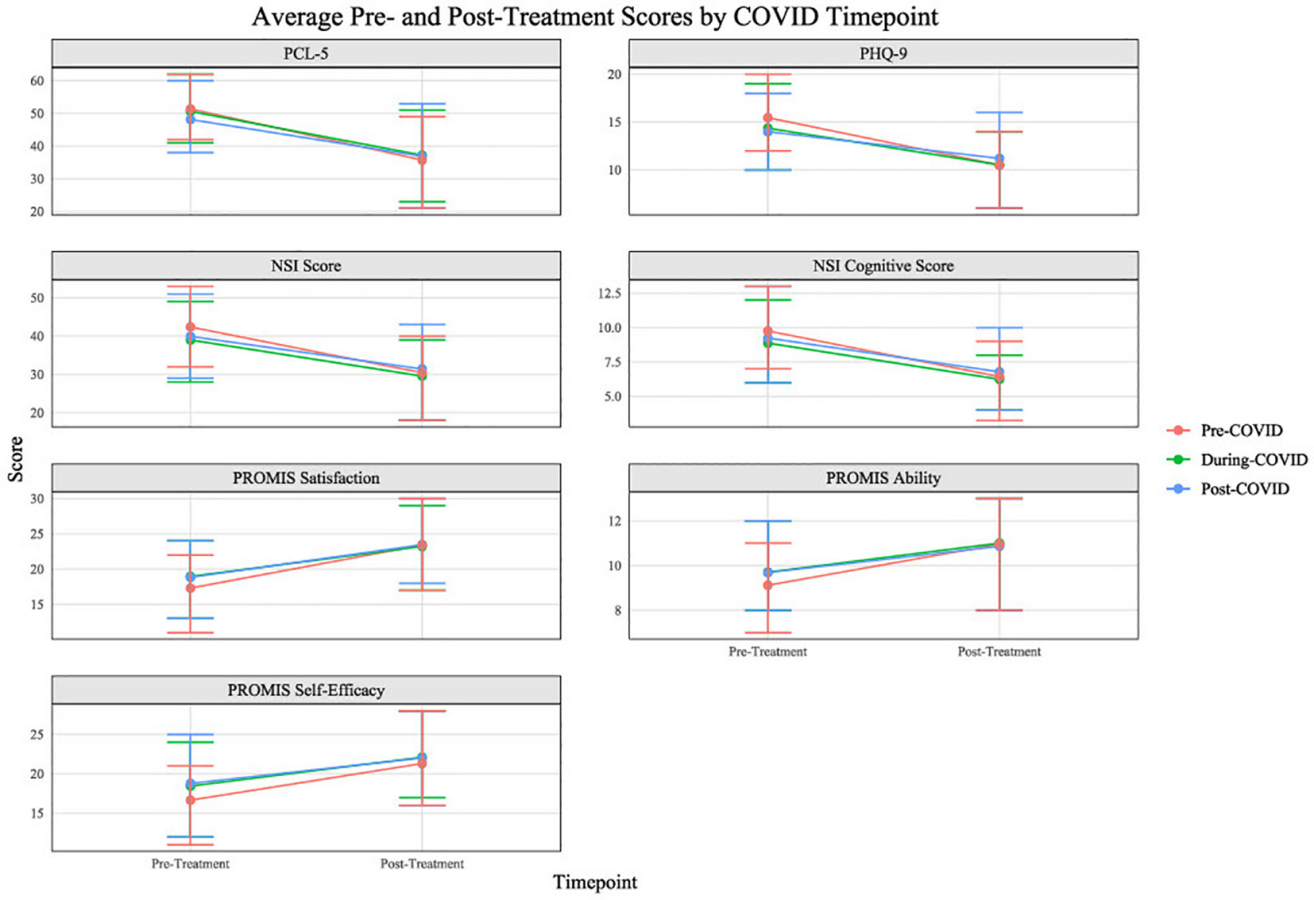
Average pre- to post-treatment measure score changes by COVID timepoint.

**Figure 5:**
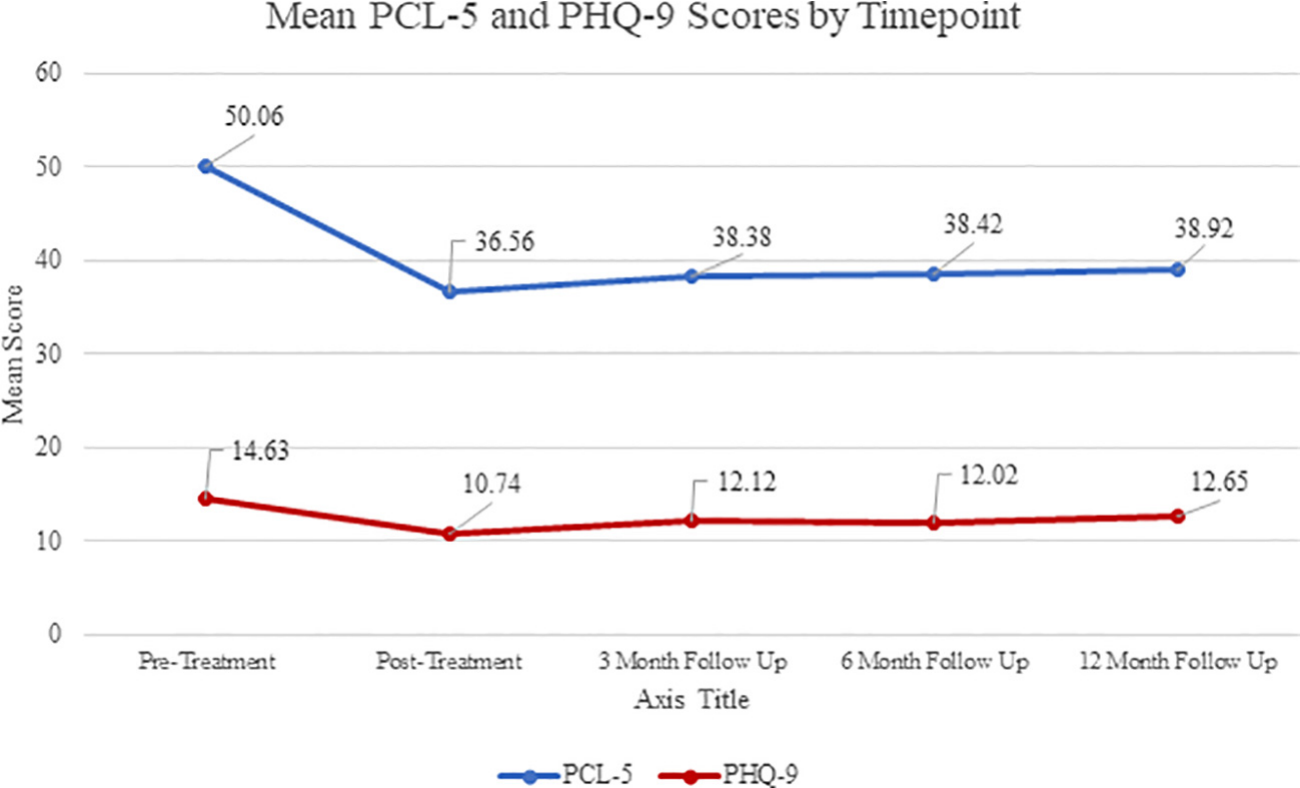
PCL-5: Pre-Treatment n=2431, Post-Treatment n=2141, 3 Month n=751, 6 Month n=578, 12 Month n=413; PHQ-9: Pre-Treatment n=2474, Post-Treatment n=2161, 3 Month n=717, 6 Month n=540, 12 Month n=415.

Patients who attended the program during the COVID-19 pandemic experienced a decrease in pre- to post-treatment score changes compared to patients who attended prior to the beginning of the pandemic (**Table 5**; **Figure 4**). Additionally, follow-up measures were collected from participants at 3-month, 6-month, and 12-month. Mean self-reported scores for the PCL-5 and the PHQ-9 are presented in **Figure 5**. Despite a slight increase in PTSD symptoms and depression scores at 3-month follow-up, scores remain significantly lower at all subsequent time points than at pre-treatment.

**Table 5:** Pre- to Post-Treatment Measure Score Changes by Time-Interval.

	Pre-COVID	During-COVID	Post-COVID	*F*	*P*
	*M (SD)*	*M (SD)*	*M (SD)*		
PCL-5 *(N=786, 578, 709)*	-15.52 (16.49)	-12.77 (16.43)	-11.33 (17.01)	12.24	< 0.001^a,b^
PHQ-9 *(N=791, 608, 725)*	-4.99 (5.53)	-3.71 (5.71)	-2.76 (5.68)	29.72	< 0.001^a,b,c^
NSI Score *(N=755, 563, 693)*	-12.24 (14.75)	-8.61 (12.79)	-8.16 (13.05)	19.24	< 0.001^a,b^
NSI Cognitive Score *(N=301, 234, 222)*	-3.15 (3.82)	-2.78 (3.66)	-2.35 (3.79)	3.01	0.050
PROMIS Satisfaction *(N=793, 607, 730)*	6.10 (8.19)	4.39 (7.54)	4.53 (7.41)	11.23	< 0.001^a,b^
PROMIS Ability *(N=791, 610, 742)*	1.77 (3.31)	1.25 (3.08)	1.13 (3.15)	8.52	< 0.001^a,b^
PROMIS Self-Efficacy *(N=781, 591, 718)*	1.13 (3.15)	3.45 (9.73)	2.99 (9.47)	6.79	0.001^a,b^

a = significant difference at the 0.05 level between Pre-COVID (September 2015 to February 2020) and During-COVID (March 2020 to August 2022) mean change scores,^b^= significant difference at the 0.05 level between Pre-COVID and Post-COVID (September 2022 to July 2024) mean change scores, ^b^= significant difference at the 0.05 level between During-COVID and Post-COVID mean change scores.

One-way ANOVA tests conducted to determine if gender or race impacted treatment outcomes yielded non-significant findings. A linear regression conducted to determine if the age of the participant could predict pre- and post- treatment score changes for the PCL-5 and PHQ-9 did not find any statistically significant results for the change in score for the PHQ-9. Although, results for the PCL-5 were statically significant, age accounted for less than 1% of the variability in PTSD scores (*R^2^ =* 0.004*, F*[1, 2071] = 7.60, *p =* 0.006).

A one-way ANOVA test comparing the effect of different treatment types on the change in pre- to post-treatment PHQ-9 scores revealed statistically significant differences between several treatment groups, *F*(5, 2112) = 4.08, *p* = 0.001. A *post-hoc* comparison using the Games-Howell test indicated that participants receiving CBT exhibited a significantly greater reduction in PHQ-9 scores compared to those receiving UP (M = -3.01, *p* = 0.003). Similarly, compared to UP, those receiving CPT and PE evinced greater reductions in PHQ-9 scores (CPT: M = -1.78, *p* = 0.01; PE: M = -1.77, *p* = 0.01). The effect of different treatment types on the change in pre- to post- treatment PCL-5 scores was not significant.

The independent samples *t*-test revealed that the presence of a support person during treatment may impact treatment outcomes for both PTSD symptoms, *t*(2071) = -4.05, *p* < 0.001, *d* = -0.19, and depressive symptoms, *t*(2122) = -3.93, *p* < 0.001, *d* = -0.178. Participants with, compared to those without a support person present, exhibited greater decreases in PTSD symptoms (M = -14.40, SD = 16.39 vs. M = -11.29, SD = 17.22, resp.), as well as greater decreases in depressive symptoms (M = -4.22, SD = 5.72 vs. M = -3.20, SD = 5.64, resp).

## Discussion

This report describes a two-week massed treatment program for veterans and active duty service members struggling with symptoms of PTSD, anxiety, depression, TBI, prolonged grief, and co-occurring substance use. Although a diagnosis of PTSD is not required for participation in the program, it is a primary concern reported by participants with 88% of all participants seeking evaluation or treatment for PTSD and 2.5% seeking evaluation or treatment for PTSD and TBI.

Consistent with prior literature (12), providing trauma-focused treatment via a massed protocol was effective in the long-term reduction of trauma-related symptomatology in veteran and military populations. Such a massed protocol has several advantages including a low dropout rate, as evinced by the overwhelming number of our participants completing the program. Consistent with established literature (9, 64), patients with PTSD and comorbid sequelae from TBI also benefited from trauma-focused care. Expectedly, these gains extended into other quality-of-life functioning (e.g., 65).

In an effort to better understand who may benefit from a massed protocol, and how the benefit is conferred, analyses investigated subsets of the data. Initial analyses indicated that demographic variables did not play a statistically significant role in treatment outcomes. Further analyses demonstrated statistically significant differences among the modality of individual therapy (i.e., UP vs. PE vs. CPT vs. general CBT). On one hand, these findings may indicate that depressive symptom cluster in patients seeking trauma-focused treatment may respond to evidence-based treatments for trauma such as PE or CPT, given the substantial overlap between PTSD and depression diagnostic criteria. On the other hand, these findings do not surpass the ten points change that the National Center for PTSD considers clinically significant, therefore extrapolation of their relevance beyond the statistical context should be tempered. Similarly the differences by time period (pre-, during- and post-COVID) need to be tempered as they meet the threshold for statistical significance but do not cross the clinically meaningful thresholds for the measures. While a component analysis is outside the scope of this manuscript, further investigation into the reduction of change scores over time is important to ensure that programmatic changes are having the desired and expected effects, particularly because this trend persists for several measures.

The expected effects were seen for the MH track providing greater benefits for PCL-5 and PROMIS Ability scores, but the conversely expected effects – NSI improving more for TBI track patients than MH patients was not evinced. Nor were, curiously, the differences in change score for symptoms of depression. This lack might be accounted for by the programming (i.e. Cognitive Health for NSI and wellness programming and/or DBT for PHQ-9) or the significant overlap among the measures' items and symptom clusters.

In line with extant findings (66) having a support individual engage with the adjunctive programming was associated with better symptom improvement over the course of the program. Interpreted conservatively, this finding could reflect the benefit of having strong social supports, measured here by the presence of an individual dedicated enough to one's recovery to commit to participate in psychoeducation and skills training for the benefit of the participant. More liberally, this finding could demonstrate some direct support for the burgeoning investigations (6) of benefit of supportive individuals in patients' trauma-focused treatment (50).

Given the high completion rate and strong outcome data, this model should be considered as an option for military service members struggling with symptoms that lead to lower functioning and poorer quality of life. Findings add to the emerging literature that massed treatments for trauma are well tolerated and show long-term benefits in this population (12).

## Future opportunities

### Indigenous veterans and service members

The Home Base ICP lends itself naturally to innovation, specifically as it relates to underserved populations. In 2023, Home Base piloted a program for Native American and Native Hawaiian veterans and service members. According to the Department of Veterans Affairs, American Indians and Alaska Natives serve in the military at proportional rates higher than any other group (67), and indigenous and rural veterans face unique challenges, including elevated suicide rates (68). Poverty, infrastructure challenges, and limited access to specialty care make it exponentially more difficult for those impacted by the effects of traumatic stress disorders, depression, anxiety, and substance use issues to attain treatment. Home Base has developed relationships with multiple organizations to build out a program that serves the unique needs of this population. Though piloted in Boston, this program plans to launch as a mobilized program on tribal land in the Southwest, providing nearly 30 hours of personalized, culturally sensitive treatment, to include individual and group therapy, wellness activities, and peer-to-peer support. Upon completion of the program, case management services will be provided to ensure a smooth transition to care in home communities.

### Acknowledgement of any conception or methodological constraints

The Home Base ICP data comprise self-report measures, rather than structured clinical interviews or controlled assessments, largely because of time limitations and regulatory limitations to administering clinical assessments, such as the Clinician-Administered PTSD Scale for DSM-5 (CAPS-5), across state lines prior to or after the program. While the PCL-5 is a well-validated measure and can be used to identify a provisional PTSD diagnostic status, quantify the severity of symptoms, and detect clinical change, it does not have a Criterion A component, which leaves into question how many patients meet criteria for PTSD at the pre- and post-treatment timepoints (69). It is also worth noting that with a mean score of 39 on the PCL-5 at post-treatment, many participants may still experience PTSD symptoms at the end of treatment. As this is a clinical program and not a treatment trial, no comparison group exists for participants which opens threats to validity (*e.g.* selection bias, participant response bias) and could limit generalizability. This manuscript presented the results of a completer analysis, rather than an intent-to-treat analysis used in randomized controlled trials. Completer analyses can lead to inflated treatment outcomes. Participants with missing data were excluded from analyses, which could also artificially inflate treatment outcomes. The aim of the manuscript is to outline and evaluate this specific program so that others can advance from both our successes and limitations. Additionally, the program is funded by grants, partnerships, and philanthropy, and therefore significantly reduces barriers to care by providing treatment and transportation at no cost to participants. However, this could have implications on program development based on funding requirements, such as strict guidelines related to treatment delivery, as well as limiting the replicability due to the unique context (financially, institutionally, collaboratively, etc.) in which this clinic operates. Finally, the program has approximately 80 clinical and administrative staff members in order to serve 24 participants every two weeks, which may risk challenges with consistency across providers and program dissemination. Further research regarding consistency of treatment and assessment of delivery is warranted.

## Data Availability

The raw data supporting the conclusions of this article will be made available by the authors, without undue reservation.
